# Neuroinflammation and M2 microglia: the good, the bad, and the inflamed

**DOI:** 10.1186/1742-2094-11-98

**Published:** 2014-06-03

**Authors:** Jonathan D Cherry, John A Olschowka, M Kerry O’Banion

**Affiliations:** 1 Department of Pathology and Laboratory Medicine, University of Rochester School of Medicine and Dentistry, Rochester, NY 14642, USA; 2 Department of Neurobiology & Anatomy, University of Rochester School of Medicine and Dentistry, Rochester, NY 14642, USA

**Keywords:** alternative activation, M2, microglia, neuroinflammation

## Abstract

The concept of multiple macrophage activation states is not new. However, extending this idea to resident tissue macrophages, like microglia, has gained increased interest in recent years. Unfortunately, the research on peripheral macrophage polarization does not necessarily translate accurately to their central nervous system (CNS) counterparts. Even though pro- and anti-inflammatory cytokines can polarize microglia to distinct activation states, the specific functions of these states is still an area of intense debate. This review examines the multiple possible activation states microglia can be polarized to. This is followed by a detailed description of microglial polarization and the functional relevance of this process in both acute and chronic CNS disease models described in the literature. Particular attention is given to utilizing M2 microglial polarization as a potential therapeutic option in treating diseases.

## Background

For the better part of a century, the function of microglia in the central nervous system (CNS) was a topic wrapped in controversy. Originally identified by Franz Nissl in 1899 as ‘Stabchenzellen’ or rod-like cells, and further classified by Pío del Río Hortega in 1919, these cells were determined to be a distinct non-neural and non-astrocytic population [1]. Furthermore, Hortega’s observations suggested a capacity for phagocytosis, indicating that these cells were more than just space filler or connective cells between neurons. Not all shared Hortega’s ideas. This included his mentor, the father of modern neuroscience, Ramón y Cajal, who urged Hortega not to publish and subsequently fired Hortega [2]. This early turmoil set the tone for decades to follow, during which the topics of microglial functions and origins were extensively debated [3]. However, beginning in the early 1980s, newer technology and ideas began to reveal the true nature of microglia as the brain’s resident immune cell. Even though we are starting to understand what microglia are capable of, many questions still remain. In particular, there is much to be learned about the plastic nature of these cells and the functions served by different microglial phenotypes.

In agreement with Hortega’s original description, microglia have been classically described to exist in two states, resting and activated. However, this binary definition has since been revised to make way for more complex ideas. Microglia in the healthy CNS are not truly ‘resting’. Two-photon microscopy has shown microglia to be engaged in environmental surveillance, constantly sampling areas around them in efforts to maintain homeostasis [4]. Once microglia encounter a substance that they sense is foreign or indicative of harm, they enter an ‘activated’ state. As macrophage-like cells of the brain, one of the main roles of activated microglia is that of regulating CNS innate immunity and initiating appropriate responses, such as inflammation. In the brain, this inflammatory response, termed neuroinflammation, is a fundamental response generated to protect the CNS; however, uncontrolled or prolonged neuroinflammation is potentially harmful and can result in cellular damage. This is particularly relevant to neurodegenerative diseases, which are typified by evidence of microglial activation and neuroinflammation [5]. This makes microglial activation an attractive target to study as part of disease pathogenesis.

The term activation is an oversimplification of a range of different ‘activated’ states. It is now recognized that activated microglia can exist broadly in two different states [6]. The first is classical activation, which is typified by the production of inflammatory cytokines and reactive oxygen species, while the second is a state of alternative activation, in which microglia take on an anti-inflammatory phenotype involved in wound repair and debris clearance [7]. It stands to reason that during neurodegenerative disease, where neuroinflammation is a prominent feature and potential contributor to disease, these alternatively activated microglia would be beneficial in resolving pathology.

The presence of multiple activation phenotypes for microglia is a relatively new concept that is only starting to gain momentum. Therefore, the amount of data is still sparse on the roles they play. However, the activation status of peripheral macrophages has been an area of interest ever since Stein and colleagues observed that interleukin 4 (IL-4) induced macrophages to express the mannose receptor [8]. Such a phenotype was previously unseen and was therefore designated ‘alternative’. Since then, multiple laboratories have characterized and classified ‘unique’ activation states, leading to a somewhat convoluted set of nomenclature. However, recent evidence suggests that the *in-vitro* data originally used to identify macrophage phenotypes does not accurately model the complex tissue environment and the original descriptions are somewhat simplistic [9]. Moreover, unlike the periphery, in which these cells have been studied for more than 20 years, we are just beginning to closely examine these complicated activation states in the CNS. Therefore, a deeper understanding of the heterogeneity and different phenotypes of microglia is needed; assumptions that information gleaned in the periphery will translate to the brain may not be correct.

## Classical versus alternative activation

Neuroinflammation, and, to the same degree, all inflammation, is a fundamental immune response designed to protect the body from harm, arising from both endogenous and exogenous sources. Being the sentinel immune cell of the brain, microglia are tasked as the first responders to infection or tissue injury and initiating an inflammatory response. Using a full array of immune receptors, such as toll-like receptors (TLRs), nucleotide-binding oligomerization domains (NODs), NOD-like receptors, and many scavenger receptors [10,11], microglia (as well as other CNS cells, such as astrocytes) are able to recognize harmful stimuli and respond by producing inflammatory cytokines such as TNFα, IL-6, IL-1β, interferon-γ (IFNγ), and several chemokines [12]. This cytokine production is essential for the polarization of microglia into what has been termed a classically activated, ‘M1’, state [13]. This term parallels the Th1 terminology used for T cells, and underscores the close relationship between T cells and macrophages in the periphery. Interferon-γ produced from Th1 cells was found to be instrumental in polarizing macrophages to M1 [14]. However, the ability to produce these cytokines does not rest solely with T cells. Microglia and astrocytes have also been observed to fill this role [15,16], demonstrating, at least in part, that microglia can control their own polarization through autocrine and paracrine means. In many cases, this response is protective and is downregulated once the damage or pathogen has been dealt with; however, unregulated, long-term, or chronic inflammation can lead to tissue destruction [17].

In contrast with proinflammatory M1 cells, alternatively activated macrophages express cytokines and receptors that are implicated in inhibiting inflammation and restoring homeostasis. This includes production of IL-10 to downregulate inflammatory cells, extracellular matrix protecting proteins like YM1, ornithine, and polyamines for wound repair, and higher levels of receptors associated with phagocytosis, such as scavenger receptors [18]. Just as the Th1 cytokine IFNγ has been associated with induction of proinflammatory M1 macrophages, the Th2 cytokine IL-4 has been associated with M2, or alternative, activation. In the periphery, M2 cells are not always associated with protective functions. In addition to parasite protection, wound repair, and debris clearance, these cells are also potential key players in asthma and allergy responses [7]. However, these types of harmful reaction may not be relevant in the CNS, demonstrating a divergence between peripheral and central cells. Interestingly, it appears that when there is a lack of M2 cell differentiation in the CNS, problems can arise (this is discussed in detail later).

### Phenotype of M1 cells

To properly understand the role microglia play in neurodegeneration, understanding their phenotypes is important. The functional effects of classical activation are geared towards antigen presentation and the killing of intracellular pathogens. Therefore, upregulation of many associated receptors and enzymes reflects that purpose. For example, MHC II, CD86, and Fcγ receptors are upregulated to allow for antigen-presenting activity of microglia and increased crosstalk with other immune cells [19]. In addition, the ratio of particular cytokines has been used to identify inflammatory macrophages and this observation could extend to microglia. For example, since M1 macrophages were found to be a key source of IL-12 [20], it was suggested that IL-12^High^, IL-10^Low^ production is a simple way to distinguish inflammatory cells [21]. Another potential distinction and an important component of M1 microglia is their ability to produce reactive oxygen species and reactive nitrogen species [22]. A key microglial enzyme associated with this process is inducible nitric oxide synthase (iNOS), which utilizes arginine to produce nitric oxide [23]. However, even though it seems straightforward to identify M1 cells based on these characteristics, classifying these cells *in vivo* has proven to be more challenging, reflecting the plastic nature of microglia.

### Phenotype of M2 cells

There is not one set description or classification of M2 cells. In fact, there are many efforts to identify unique sub-groups with different functions. Division of M2 cells is based on observations that stimulation with various cytokines yields different sets of receptor profiles, cytokine production, chemokine secretion, and function [21]. Even though the profiles of these cells are diverse, the one feature that places them all in the M2 classification is that they express mediators or receptors with the capacity to downregulate, repair, or protect the body from inflammation [24].

The original alternatively activated macrophage was classified based on expression of the mannose receptor [8]; since then an assortment of different markers has been identified as ‘M2’ specific. One of the best characterized markers is the enzyme arginase 1 (Arg1) [25], which converts arginine to polyamines, proline, and ornithines that can contribute to wound healing and matrix deposition [26]. Interestingly, by using arginine, which is the same substrate used by iNOS, Arg1 can effectively outcompete iNOS to downregulate production of nitric oxide [27,28]. Thus, iNOS and Arg1 represent a relatively straightforward set of markers to follow M1 versus M2 phenotypes. Other markers used for identifying M2 cells include Ym1, a heparin-binding lectin [29,30], FIZZ1, which promotes deposition of extracellular matrix [31], and CD206, a mannose receptor [8]. A list of additional markers can been found in Table 1. Despite the benefit of having specific markers, using just one or two is limiting and ignores the overall diversity of M2 phenotypes.

**Table 1:** M2 markers

**M2 marker**	**Function**	**Expressed in murine microglia?**	**Expressed in human microglia?**	**Reference**
Arginase 1 (Arg1)	Converts L-arginine into prolines and polyamines implicated in tissue remodeling and wounding healing. It competes with iNOS for substrates.	Yes	Debated: evidence suggests it is not upregulated in human beings but others have shown elevations in the CNS.	[6,27,28,32,33]
Ym1 (Chi3l3)	A secretory lectin that binds heparin/heparan sulfate. It is proposed to prevent degradation of extra cellular matrix components.	Yes	Similar to arginase 1 as it has been seen in human beings. However, it might not be expressed in M2 microglia.	[6,30-33]
FIZZ1 (RetnlB)	Mediates interactions between sensory nerves and inflammatory cells in the lungs. It blocks nerve growth factor induced survival of dorsal root ganglion neurons.	Yes	Unknown	[31,34,35]
MRC (CD206)	Binds and internalizes/phagocytoses mannosylated ligands.	No, limited to perivascular macrophages and meninges	Observed *in vitro*.	[36-38]
CD163	Binds and internalizes hemoglobin-haptoglobin complex.	Yes	Yes	[39-41]
TREM2	The endogenous ligand is unknown, but thought be involved in debris clearance.	Yes	Yes	[42,43]
Dectin-1 (Clec7A)	Recognizes β-glucans and can lead to phagocytosis.	Yes	Observed in human macrophages but unknown whether it is expressed in human microglia.	[19,44,45]
CD301 (MGL1)	Recognizes terminal galactose and N-acetylgalactosamine. Involved in pathogen defense and related to CD206.	Yes (our observations)	Unknown	[46]

Another way to classify the function and phenotype of M2 cells is based on the cytokines that induce them. The prototypical cytokine used to first induce alternative activation was IL-4 [8]. Both IL-4 and the closely related cytokine IL-13 signal through IL-4Rα to induce a host of downstream processes that lead to potent anti-inflammatory functions, such as Arg1 upregulation, inhibition of NF-κB isoforms, and production of scavenger receptors for phagocytosis [19,47,48]. This type of activation has been classified as ‘M2a’. The main function of this response appears to be suppression of inflammation. A second state of alternative activation is based on macrophages exposed to IL-10, glucocorticoids, or TGF-β. This phenotype is classified as ‘M2c’ [21,49]. Originally, this state was described as being ‘deactivated’ but that is not a particularly useful description. Instead of having no function, ‘deactivated’ M2c macrophages appear to be involved in tissue remodeling and matrix deposition after inflammation has been downregulated [21]. A third sub-class of M2 activation has been observed following exposure to immune complexes and stimulation of TLR. This class is termed ‘M2b’ or Type II [21,50]. Of these three classes, M2b macrophages are the least understood. Interestingly, they more closely resemble M1 macrophages, owing to the lack of any M2 specific markers, such as Arg1, YM1, or FIZZ1. However, they do express the typical IL-10^High^, IL-12^Low^ M2 cytokine profile [9]. Moreover, they have higher levels of MHCII and CD86, suggesting that they retain their ability to stimulate T cells [50]. Interestingly, it appears that when M2b macrophages stimulate T cells they are biased towards a Th2 response [51]. Being able to induce Th2 T cells suggests that M2b might be a potential regulator or initiator of the M2 response in general. One additional type of M2 activated cell, the so-called tumor-associated macrophage, has recently been recognized [52]. Although tumor-associated macrophages are an area of intense research [53], they are beyond the scope of this review. An important consideration regarding M2 phenotypes is that these states were typically elucidated *in vitro* following exposure to one or two stimuli. This does not represent the complex environmental milieu seen in tissue. Therefore, some investigators have cautioned against this classification into distinct subtypes and instead propose that M2 cells should be viewed as a spectrum of phenotypes [9].

This detailed classification of M2 cells has been primarily carried out in the periphery. Whether or not this will extend to brain resident microglia is yet to be seen. However, some investigators have taken to using the M2a-c nomenclature to discuss populations of alternatively activated microglia [54]. This poses potential problems, as certain M2 markers do not appear to be expressed in the CNS (Table 1). The best example of this is the first observed alternative macrophage marker, CD206, which is only seen in perivascular or choroid-plexus-associated macrophages and not expressed by parenchymal microglia [55]. Furthermore, microglia are not macrophages that migrate into the brain, but instead are known to represent a distinct population of resident tissue mesenchymal cells that populate the CNS during early development [56,57]. Importantly, because the origins and responses of microglia and macrophages are different, the roles they play in mitigating or propagating pathology could be different as well.

## M2 microglial activation during acute neuroinflammation

In mechanical injuries like spinal cord and traumatic brain injury or other relatively acute conditions like ischemic reperfusion injury, released damage-associated molecular patterns (DAMPs) induce the innate immune system to activate and produce inflammatory cytokines and reactive oxygen species [58-60]. As previously stated, this response is not purposefully harmful. In fact, it is a necessary step in wound repair [61]. The initial proinflammatory response is to promote killing of any invading organism and remove dead cells to ‘clean’ the damaged area [62]. This response is then shifted to an anti-inflammatory state where debris clearance, extracellular matrix deposition, and angiogenesis are promoted [24]. Thus, when there is proper transition from the M1 to M2 phenotype, the damage can be efficiently repaired. However, when the proinflammatory response does not yield, the constant presence and continued production of inflammatory cytokines and reactive oxygen species can lead to cell death and further tissue damage [63].

### Spinal cord injury

One of the better-studied areas of M1 and M2 activation in the CNS is after spinal cord injury. Following the initial trauma of spinal cord injury, secondary inflammation has been identified as an important factor that leads to enhanced damage and impaired regeneration. Consistent with this, several M1 microglial secreted factors have been shown to be neurotoxic and inhibit axon extension [63-65]. Kigerl *et al.*[63] characterized the response of M1 and M2 cells both acutely and several weeks after spinal cord injury. Initially, they observed early upregulation of both M1- and M2-related proteins and mRNA species. However, three days post-injury, the M1 markers continued to rise and M2 markers were downregulated, leading to a skewed M1 profile [63]. Kigerl *et al.* suggested that the domination of M1 cells might be one of the reasons for continued damage and lack of repair. For example, in the same report, using cultured neurons, IFNγ-polarized M1-conditioned media was neurotoxic and prevented axon elongation after injury, while IL-4 treated, M2-conditioned media encouraged axon growth [63]. The beneficial functions of IL-4 appear to extend to endogenous IL-4, since IL-4^−/−^mice showed increased damage following spinal cord injury [66]. Other groups have observed similar polarization dynamics after spinal cord injury [67,68]. The positive effects of M2 cells on spinal cord injury can also be extended to the enzymatic products of Arg1 activity. For example, Cai *et al.* demonstrated that polyamines were sufficient to block the suppressive effects of myelin and myelin-associated glycoprotein on dorsal root ganglion neuron regeneration [69].

### Traumatic brain injury

As with spinal cord injury, the damaged tissue environment after traumatic brain injury heavily favors activation of proinflammatory M1 microglia [70]. Several studies demonstrating a protective effect of anti-inflammatory treatment indicate that the inflammatory response after trauma contributes to the ensuing damage [71-73]. The aftermath of traumatic brain injury results in a mixed profile of activated microglia and macrophages exhibiting a range of phenotypes [74]. However after one week, just like with spinal cord injury, the concentration of Arg1^+^ cells decreased to non-detectable levels [74], and other studies showed the presence of M1 microglia and proinflammatory cytokines weeks to months after traumatic brain injury [74-76]. This suggests that M1 microglia are the dominant phenotype and M2 cells are not present to repair damage. To highlight the importance M2 microglia might play after traumatic brain injury, it was observed in aged mice where the M2 response is impaired (discussed in a later section) that lesion size was increased relative to young animals with a more functional M2 response [70]. The M2 microglia observed during traumatic brain injury do seem to possess beneficial phenotypes that can mitigate damage associated with traumatic brain injury. For example, M2 cells recruited around sites of intracranial hemorrhage after traumatic brain injury expressed the receptor CD163, which functions in hemoglobin scavenging [39,40,77].

### Stroke

Inflammation generated by stroke and ischemic reperfusion injury is regarded as a major factor contributing to tissue damage [60] and, like spinal cord injury, the damaged tissue environment favors an M1 phenotype [78]. In addition to neutrophils, it is these M1 microglia, and, to a lesser degree, macrophages, that contribute to the inflammatory cascade and further propagate cell death beyond the initial ischemic region [79,80]. Even though the proinflammatory response is dominant, there does appear to be an anti-inflammatory signal that attempts to regulate the inflammation [78]. Consistent with the idea that an M2 response is needed to properly downregulate inflammation and initiate repair, mice that lack appropriate signals for M2 induction have worse outcomes after experimental cerebral ischemia. For example, mice lacking either IL-4 or IL-10 show increased infarct volume and worse cognitive performance following cerebral ischemia [81,82]. Additionally, deletion of galectin-3, a protein required for microglial activation, leads to a reduction in M2-associated cytokines, such as IGF-1, which results in worse pathology after stroke [83]. This highlights the importance of M2 cells in mitigating and repairing damage.

### Controlling polarization

The experiments described above demonstrate the crucial dynamics between M1 and M2 polarization during injury state. One piece of information that comes out of this research is the critical role the environment plays in controlling the shift from classical to alternative activation. With spinal cord injury and traumatic brain injury, the domination of M1 microglia is mainly due to the high levels of proinflammatory cytokines present [60,68]. Importantly, polarized microglia are not locked in a particular state; both microglia and macrophages are plastic cell types that can be altered if the cytokine environment changes [84]. However, in many acute injuries, the continued production of cytokines like IFNγ and TNFα maintains an M1 activation state. One would therefore hypothesize that altering the environment could be used to treat injuries. To that end, in models of the injuries described previously, investigators have begun to utilize techniques to inject M2 cells directly or cause polarization indirectly. In spinal cord injury, the transplantation of mesenchymal stem cells has been shown to increase IL-4/13 and decrease TNFα levels [85]. These cytokine changes were associated with increased Arg1^+^ staining, consistent with an M2 response, and associated with downregulation of inflammation, locomotor recovery and reduced scar formation [85]. This has been shown in several other cases, where M2 cell induction appeared to alleviate spinal cord injury pathology [86-89]. This beneficial result is not limited to spinal cord injury. By targeting the PPARγ pathway with PPARγ agonists that potentially lead to induction of M2 microglia, several groups have shown efficacy in treating traumatic brain injury [71,72] and ischemia [90]. As summarized in Table 2, there are several other examples of investigators using various molecules that are now known to induce M2 polarization in order to treat CNS injuries.

**Table 2:** M2 inducing agents used in disease models

**Disease or injury**	**Treatment**	**Outcome**	**Reference**
Traumatic brain injury	Rosiglitazone	24 hours after controlled cortical impact, rosiglitazone was given intraperitoneally. Seven days later there were decreased cortical lesions and reduced glial activation. A reduction in apoptotic cells was also seen.	[72]
Ischemia	Rosiglitazone	Rosiglitazone was given orally before ischemia was induced. Treatment reduced damage in the hippocampal CA1 region and delayed neural death. Elevated levels of IL-4 and IL-13 were seen after treatment.	[90]
Spinal cord injury	Rosiglitazone	Rosiglitazone was injected intraperitoneally every 12 hours after spinal cord injury for 12 days. Treatment decreased tissue damage and significantly reduced apoptosis in damaged tissue. TNFα and IL1β reduction was also observed.	[91]
	Mesenchymal stem cell transplantation	Transplantation in an injured spinal cord resulted in elevated levels of IL-4 and IL-13 and reduced TNFα and IL-6. There was functional locomotion improvement as well as reduced scarring and more preserved axons.	[85]
	Granulocyte colony-stimulating factor	Granulocyte colony-stimulating factor was injected for three consecutive days after spinal cord injury. Enhanced Arg1 and CD206 mRNA and reduced iNOS, TNFα, and IL-1β were seen. There was also reduced NF-κB activity. No locomotor tests were performed, but the authors concluded that this is a viable method to reduce acute phase inflammation after spinal cord injury.	[89]
	Substance P	Substance P was injected intravenously immediately, 24, and 48 hours after spinal cord injury. Injected rats had reduced iNOS, IL-6, and TNFα mRNA levels and elevated amounts of Arg1 and IL-10. M2 cells were observed at the lesion site. Spinal cord lesions were significantly smaller and injected mice had improved locomotion scores.	[92]
Multiple sclerosis or EAE	IL-4	Herpes simplex virus carrying an IL-4 sequence was injected into the CNS of mice with EAE. Treatment delayed progression of disease and improved clinical score. Significant reduction in inflammation and axon degeneration was also seen.	[93]
	IL-4	Used transduced T cells carrying a retroviral gene to express IL-4 during EAE. Mice showed delayed onset and reduced severity of disease.	[94]
	IL-4	Adenoviral-vector-carrying IL-4 was injected into cerebrospinal fluid of mice induced to have EAE. Reduced clinical score and improvement in several neurophysiological parameters were seen in injected mice.	[95]
	IL-10	Used adult neural stem cells engineered to express IL-10. Intraperitoneal injection during EAE reduced CNS inflammation and lessened demyelination. Additionally there was enhanced remyelination.	[96]
	Glatiramer acetate	Glatiramer acetate is a synthetic peptide shown to be beneficial in treating relapsing and remitting multiple sclerosis. It shifts the CNS environment from Th1 to Th2 and induces secretion of anti-inflammatory cytokines.	[97]
	IL-33	Intraperitoneal injection of IL-33, 12–20 days after induction of EAE, reduced inflammatory cytokines and improved clinical scores. Elevated M2 cells were also seen around lesions.	[98]
Alzheimer’s disease	Glatiramer acetate	Vaccination with glatiramer acetate caused a reduction in amyloid β plaques in APP/PS1 mice. The effect is thought to be mediated in part by glatiramer acetate inducing IL-4, which can counteract the effect of amyloid β on microglia. Glatiramer acetate vaccination also reversed cognitive decline.	[99]
	IL-4	Intracerebral injection of IL-4 and IL-13 reduced amyloid β plaque load in APP23 mice with Alzheimer’s disease. The decrease was accompanied with improved cognition and upregulation of Arg1 and YM1 positive M2 cells.	[32]
	IL-4	AAV carrying an IL-4 sequence was injected intrahippocampally in 3-month-old APP/PS1 mice. Five months later there was a reduction in amyloid β plaques, improvement in the Morris water maze memory task, and elevated neurogenesis.	[100]
	IL-10	AAV expressing IL-10 was injected intrahippocampally in 3-month-old APP/PS1 mice. Unlike previous experiments with IL-4, AAV-IL10 did not clear amyloid β plaque at 5 months. However, neurogenesis was improved.	[101]
	DSP-8658	DSP-8658 (a PPAR γ/α agonist) treatment was able to increase microglial uptake of amyloid β in APP/PS1 mice. The mechanism of action was thought to be via CD36 upregulation on microglia.	[102]
	Bexarotene	Bexarotene (retinoid X receptor agonist) in APP/PS1 and APP/PS1-21 mice with Alzheimer’s disease led to reduced amyloid load 14 days after oral treatment. The plaque reduction was associated with improved order habituation behavior.	[103]

AAV, adeno-associated virus; APP, amyloid precursor protein; CNS, central nervous system; EAE, experimental autoimmune encephalitis.

## M2 microglia in chronic neuroinflammation

As mentioned in the previous section, the inflammatory response needs to be downregulated for proper healing to take place. In contrast with acute inflammation, chronic neuroinflammation is a long-lived, persistent response that starts with an initial inflammatory stimulus, but becomes self-propagating. Inflammatory factors produced by microglia and astrocytes can damage local tissue and, together with released DAMPs, can further increase inflammation and glial activation, leading to a vicious inflammatory cycle. This long-term inflammation can have disastrous consequences in the CNS, ranging from loss of synapses to impaired cognition and overt neurodegeneration [104-107]. This shift away from reparative responses may be due to a failed M2 response. Not only could the lack of M2 microglia fail to control inflammation; fewer M2 cells also mean lower levels of neuroprotective factors like IGF1 or brain-derived neurotrophic factor, which microglia produce. Thus the lack of an appropriate M2 response might be an important mechanism underlying neurodegeneration. Indeed, many investigators are starting to recognize the importance of M1/M2 dynamics in diseases characterized by chronic neuroinflammation.

### Experimental autoimmune encephalitis (EAE) and multiple sclerosis

Multiple sclerosis is a disease characterized by demyelination of axons as well as chronic inflammation. Multiple sclerosis exists in several forms; the majority of patients show a relapsing and remitting type of disease. These patients experience demyelination and inflammation but this resolves after some time. This process occurs multiple times during the course of disease, with each subsequent relapse being slightly worse, until they finally progress to secondary progressive multiple sclerosis [108]. The observation that there is resolution suggests that M1/M2 dynamics might be relevant for this disease. Although the initial cause of inflammation is not clear, it has been observed that T cells, specifically Th1 and Th17 cells, are important contributors to multiple sclerosis pathology [109]. As previously stated, Th1 cell-secreted IFNγ is a potent inducer of M1 cells, suggesting during the active phase that microglia are skewed towards M1 activation. Although T cells regulate the response, microglia and macrophages are the effector cells. Several groups have begun to examine these dynamics *in vivo* using the multiple sclerosis animal model experimental autoimmune encephalitis (EAE).

An environment dominated by inflammatory cytokines favors polarization to M1 cells and inhibits an M2 switch. The consequences of this inhibition are not fully understood, but during EAE induction and progression, inflammatory factors have the potential to prevent recovery [110]. Elevated levels of inflammatory cytokines are also observed in human multiple sclerosis [105]. It is believed that inflammation contributes to axonal demyelination owing to neurotoxic cytokine effects on oligodendrocytes or inhibition of oligodendrocyte precursor cell proliferation and maturation [110]. This places M1 cells as key contributors to multiple sclerosis pathogenesis. Indeed, mice lacking IL-4 or IL-4Rα showed significantly worse EAE pathology [111]. The importance of IL-4 in EAE is also supported by observations that transduction with an IL-4 expressing viral vector reduced the symptoms of EAE [93,94]. Even though IL-4 has actions on other CNS cell types, its most potent effect is the induction of M2 microglia. Additionally, other M2 promoting cytokines, such as IL-33 [98] and IL-10 [96], have been shown to reduce the amount of demyelination [96] and improve clinical scores [98]. It is important to note that these EAE models were of the chronic variety, as opposed to other EAE models that only display a transient pathology. This demonstrates that altering the pro-M1 environment to one more conducive to M2 generation has beneficial effects in chronic diseases (Table 2). The mechanism behind the beneficial effects of M2 cells can be attributed to their production of neurotropic mediators that support remyelination and regeneration. Factors such as IGF1, PDGFα, TGFβ, and SPP1 are all upregulated in microglia during the recovery phase of disease [112].

The beneficial effect of environmental modulation favoring M2 polarization can also be seen in human beings. The FDA-approved drug glatiramer acetate (GA), which has been shown to be useful in treating relapsing and remitting multiple sclerosis, works by inducing a Th1 to Th2 shift, resulting in the production of anti-inflammatory cytokines [97]. Even though changing the environment seems to be beneficial in alleviating symptoms for the relapsing and remitting type of multiple sclerosis, the majority of patients ultimately experience progressive disease, suggesting that the environment is not the only factor in controlling inflammation. Since multiple sclerosis is a chronic disease that takes many years to progress, the continuous long-term activation of microglia has the potential to alter microglial function, either by making them less responsive to anti-inflammatory signals or less adept at phagocytosis. This potential failure of microglia to perform their proper function is also shared by other neurodegenerative diseases characterized by persistent, long-term inflammation. One of the best examples of this is Alzheimer’s disease.

### Alzheimer’s disease

The idea that microglial activation states could impact Alzheimer’s disease has recently gained momentum. Alzheimer’s disease is the most common form of dementia and is characterized by the presence of neurofibrillar tangles of hyperphosphorylated Tau and extracellular deposits of the peptide amyloid β (Aβ), forming neuritic plaques. Another key feature of Alzheimer’s disease is the presence of prominent neuroinflammation [113]. Interestingly, Aβ itself has been shown to have proinflammatory properties when injected into the CNS [114,115]. Amyloid β can bind to several innate immune receptors present on microglia, such as TRL2 [114], TRL4 [116], TLR6 [116], and CD14 [117], all of which can lead to activation when triggered. To that end, microglia surrounding Aβ plaques show elevated production of inflammatory factors [118]. The inflammatory nature of amyloid has been recognized as a potential mechanism of disease progression. Amyloid β is generated from a parent protein called the amyloid precursor protein (APP), which is cleaved in two steps by the enzymes β then γ secretase, leading to release of the Aβ fragment [119]. This is a normal physiological process that is modified in the disease state. Interestingly, inflammation can promote accumulation of Aβ by elevating APP levels and the activity of cleavage enzymes [120]. These observations have led to the inflammatory cascade hypothesis of Alzheimer’s disease, which states that Aβ deposition induces neuroinflammation, which in turn generates more Aβ, resulting in a vicious cycle [120].

There are several ways for Aβ to be removed from the brain. Amyloid β can be directly shuttled out of the brain via protein complexes such as LRP1 and apolipoprotein E, which can bind extracellular Aβ and transport them to the blood brain barrier, where they are then shuttled to the other side [121]. Additionally, new observations suggest the existence of an alternate removal pathway, where extracellular Aβ in CNS interstitial fluid is moved into the cerebral spinal fluid in what is named the ‘glymphatic system’ [122]. Another way to clear Aβ is via phagocytosis and degradation by resident CNS immune cells, such as microglia [123], astrocytes [124], and possibly neurons [125]. This particular clearance pathway showcases the Janus face nature of microglia in that even though microglia are a primary source of inflammatory factors, they also represent a crucial element for removal of harmful material in the CNS [123]. Thus, the failure of microglia to carry out homeostatic functions possibly underscores one mechanism of increased Aβ accumulation during disease.

When Aβ is injected into the rat CNS, microglia are observed to contain the injected peptide, demonstrating their ability to take up Aβ [126]. However, *in-vitro* evidence suggests that this phagocytic ability is inhibited during disease [127]. The ability of microglia to phagocytize Aβ may depend on their phenotype. For example, *in-vitro* treatment of microglia with the pro M1 activator lipopolysaccharide inhibited microglial phagocytosis of Aβ [127]. Other proinflammatory cytokines, such as IFNγ and TNFα not only inhibited uptake of Aβ, but also prevented internalized Aβ degradation [128,129]. This demonstrates that M1 microglia might be less able to properly take up and degrade Aβ. While M1 microglia appear to be impaired in their ability to remove Aβ, M2 microglia have been demonstrated to be efficient phagocytes. Treatment with the pro M2 activating cytokine IL-4 can effectively block lipopolysaccharide-induced inhibition of Aβ phagocytosis [127] and similar data have been obtained for IL-10 [129]. This effect also extends to degradation of the internalized Aβ. Treatment with IL-4 or macrophage colony-stimulating factor can lower the pH of the phagosome and lysosome and allow for more efficient degradation of Aβ [130,131]. These *in-vitro* M1 versus M2 observations are further supported by an ever-increasing set of *in-vivo* data, as detailed next.

It appears that one of the reasons for Aβ accumulation in Alzheimer’s disease is the failure of microglia to react properly. As mentioned, inflammation limits the phagocytic potential of microglia [127], but does this occur *in vivo*? Early on in disease pathogenesis, there does seem to be an attempt to clear Aβ. Jimenez *et al.*[132] observed at 6 months, when Aβ begins to accumulate in the APP/PS1 Alzheimer’s disease mouse model, that there were YM1^+^ cells present in the CNS; however, by 18 months YM1 mRNA levels decreased and there was a massive upregulation in inflammatory factors, suggesting a switch from M2 to M1 as pathology worsened [132]. This is consistent with the idea that microglia become less responsive to M2 induction signals as they age, perhaps owing to an age-associated decrease in IL-4Rα levels [133]. Correspondingly, in older, non-diseased mice there is downregulation of receptors associated with Aβ engulfment, such as scavenger receptor A and the Aβ degradation enzymes Nep, IDE, and MMP-9 [134]. These observations suggest that the Aβ induced inflammatory environment, combined with age-associated effects on microglia, lead to a situation where M1 cells predominate and microglia lose the ability to switch phenotypes and mitigate damage.

Several groups have utilized animal models of Alzheimer’s disease to demonstrate that altering the microglial activation state can be beneficial (Table 2). As previously mentioned for multiple sclerosis, GA is a promising molecule that can alter the inflammatory environment by recruiting Th2 T cells to the CNS and induce production of IL-4. Since it has shown positive benefits in patients with multiple sclerosis, there is the potential that GA could be a useful Alzheimer’s disease treatment. Data from Michal Schwartz’s group demonstrated that GA treatment leads to increased Aβ clearance and elevated levels of neurotropic cytokines such as IGF-1 [99]. The phenotype of microglia after treatment was similar to those treated with IL-4, suggesting that the GA effect could be IL-4 (and subsequently M2) dependent [135]. PPARγ activation is another approach that can robustly induce the polarization of M2 microglia and may be a promising therapy for Alzheimer’s disease. Several groups have observed that prolonged treatment with PPARγ agonists can reduce Alzheimer’s disease pathology, demonstrating its potential therapeutic efficacy [102,136,137]. In addition to reducing plaques, the PPARγ agonist pioglitazone increased mRNA levels of the M2 marker YM1 [137] as well as the scavenger receptor CD36 [102]. Recently, activation of the retinoid X receptor (RXR), which forms a heterodimer with PPARγ, was implicated as a promising therapeutic treatment for Alzheimer’s disease. Landreth’s group [103] demonstrated that treatment with the FDA-approved RXR agonist bexarotene reduced CNS Aβ levels and improved cognition in an Alzheimer’s disease mouse model [103]. Interestingly, PPARγ treatment has been demonstrated to polarize human monocytes to an M2 state [138], which further supports the idea that manipulating proinflammatory M1 microglia to an M2 phenotype is a potentially viable therapeutic option.

In addition to pharmacological methods to induce M2 activation, direct use of anti-inflammatory cytokines can lead to Aβ removal. As previously stated, the definitions for multiple phenotypes of alternatively activated macrophages originate from the periphery and therefore might not be completely applicable to the CNS. However, microglia treated with different anti-inflammatory cytokines exhibit unique activation states and functions. This topic is actively being pursued, in order to better understand differences between states [54]. For the sake of simplicity and a point of reference, we will refer to the activation states that are known for peripheral macrophages. TGFβ is a cytokine with potent anti-inflammatory properties that polarizes microglia to an M2c phenotype. Importantly, mice overexpressing TGFβ have reduced plaque loads [139] and mice deficient for TGFβ signaling showed elevated pathology [140]. *In-vitro* cultures also confirm that TGFβ treatment can enhance microglial uptake of Aβ [141]. Interleukin-4, the prototypic M2 inducing cytokine, has been shown in several cases to mitigate Alzheimer’s disease pathology. Acute injection of 100 ng of IL-4 decreased Aβ levels in just a few days [32]. The Aβ decrease was correlated with an increase in pro Aβ phagocytic and degradation enzymes CD36 and neprilysin that colocalized to YM1 and Arg1^+^ M2 cells. Using an adeno-associated virus type 2 vector to provide sustained IL-4 expression, Kiyota *et al.* observed a reduction in gliosis, decreased Aβ, and improved spatial memory [100]. Interestingly, this same group attempted to replicate these results with IL-10 and only observed an increase in neurogenesis [101]. This discrepancy between M2-inducing cytokines suggests that different subtypes of alternatively activated microglia have unique functions. Interleukin-4-induced M2a microglia seem to be better in terms of engulfing Aβ, while IL-10-induced M2c microglia might play more of a supportive function.

Interestingly, and somewhat surprisingly, several groups, using both immunohistochemistry and ELISA as measurements, have observed that injection of inflammatory cytokines can also result in decreased Aβ. Work from our laboratory, in which the APP/PS1 Alzheimer’s disease mouse [142] was crossed with a mouse that conditionally expressed IL-1β, demonstrated that four weeks of sustained inflammation led to decreased Aβ plaque deposition as opposed to enhanced pathology [143]. Other groups have observed similar effects with different proinflammatory mediators and cytokines, such as lipopolysaccharide, IFNγ, TNFα, and IL-6 [144-147]. At first, this does not seem consistent with the *in-vitro* observations of inflammatory cytokines impairing Aβ clearance; however, one important distinction is that the *in-vitro* experiments exist in a closed system. Single treatments with cytokines *in vitro* impair phagocytic functions of microglia but do not take into account other cells and how they react to the inflammatory state *in vivo*. As previously mentioned, there is an established pattern of immune cell activation during inflammation. Initially there is a proinflammatory response, which gives way to an anti-inflammatory response that mitigates and repairs damage. Cells capable of secreting Th2 cytokines, such as T cells [148-150] and mast cells [151,152] migrate to the inflamed area and are potential sources of anti-inflammatory cytokines. However, the presence and function of these cells during Alzheimer’s disease is still debated. Even though the activity of peripheral cells is not clear, endogenous cells like astrocytes and microglia have been observed to secrete IL-4 or IL-10 during pathological conditions [153]. This important distinction between *in-vivo* and *in-vitro* data needs to be kept in context when observing how inflammatory stimuli affect the CNS.

### M2 microglia: always beneficial?

Not all share the idea that alternatively activated microglia are a beneficial cell type. As was the case with Hortega almost a century ago, the function of microglia is still a debated issue. However, the discussion now centers on the relative contributions of different microglial phenotypes and whether or not they are truly beneficial. Instead of viewing M2 microglia as alternatively activated, some believe that this cell type resembles a deactivated population that actually loses proper function. In a study by Chakrabarty *et al.*[154], an AAV vector carrying IL-4 was injected into the CNS of the TgCRND8 Alzheimer’s disease mouse model. Contrary to previously published studies [100], an increase in amyloid pathology was seen [154]. The authors reasoned that this increase is due to IL-4 inhibiting microglia from properly scavenging Aβ. However, exactly why their result is so different from other *in-vivo* and *in-vitro* data is unclear.

Additionally, even though downstream products of Arg1 activity have been observed to contribute to wound repair, matrix deposition, and axon regeneration, excess polyamines can trigger inflammation [155] and macrophage recruitment [156], suggesting that Arg1 activity can be proinflammatory. This further highlights the complex nature of alternatively activated microglia and emphasizes the need to view these cells in context rather than assume they are beneficial in all circumstances.

### Alternative activation in human beings?

The recent advances in our understanding of alternatively activated microglia and their potential efficacy in treating disease have led to greater interest in translational human studies. Unfortunately, the leap to human M2 cells is not without its own problems. As noted in Table 1, several commonly used markers, such as Arg1 and YM1 are not expressed in human myeloid cells [33], which limits the ability to identify distinct human microglial phenotypes. However, other markers appear to be consistent. CD163-positive cells have been observed in stroke and multiple sclerosis, providing a means to identify M2 microglia in human diseases [39,157]. Furthermore, mutations in TREM2, a molecule implicated in both human and murine M2 microglial function, are associated with increased risk of Alzheimer’s disease [158]. In relation to this idea, levels of M2-inducing molecules, such as resolvin D1 [21,159] and IL-10, were reduced in patients with Alzheimer’s disease [160]. Moreover, resolvin D1 levels significantly correlated with worse Mini-Mental State Examination scores [160], suggesting that the lack of factors to induce M2 polarization has potential functional relevance in human disease. To that end, efforts have been taken to examine different populations of M1 or M2 markers in human Alzheimer’s disease patients. Using several different mRNAs for M2 markers, two different populations can be identified: Alzheimer’s disease brains that are skewed towards M1 and those with an M2a bias [161]. Furthermore, the different populations are associated with different disease stages. In what appears to be early Alzheimer’s disease, there is an M1 bias, while patients with later stage Alzheimer’s disease have an M2a bias. This suggests a potential functional relevance of different microglia populations. However, whether the transition from M1 to M2a is related to disease progression or is simply a response to the enhanced pathology is yet to be understood. Obviously, more work is needed to determine just what these populations represent.

### Conclusion

**Figure 1:**
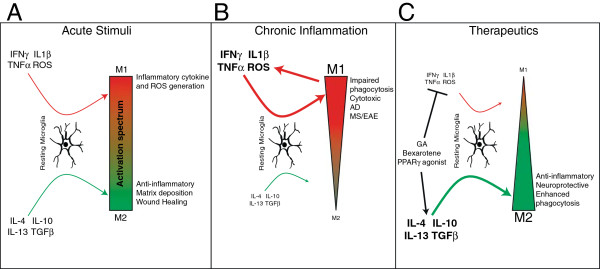
**Working model of microglial polarization. (A)** A variety of cytokines are able to polarize microglia to unique phenotypes. These phenotypes constitute a spectrum as opposed to two binary states. Depending on the stimulus, microglia can be polarized towards one end of the spectrum and be more M1- or M2-like. **(B)** Upon prolonged or chronic inflammation, an overabundance of inflammatory cytokines skews microglial polarization towards the M1 phenotype. M1 microglia, in turn, produce additional inflammatory cytokines, generating a cycle that further induces inflammation and maintains the M1 state. This skewed population of M1 microglia exhibits impaired phagocytosis and is cytotoxic in, for example, Alzheimer’s disease and multiple sclerosis. **(C)** To treat neuroinflammatory diseases, certain therapeutic agents, such as glatiramer acetate, bexarotene, and PPARγ agonists, have been used. These treatments have been shown to inhibit inflammation as well as induce M2 activation, resulting in reduced disease severity. A more complete list of such therapeutic agents can be found in Table 2. AD, Alzheimer’s disease, EAE, experimental autoimmune encephalomyelitis; GA, glatiramer acetate; MS, multiple sclerosis.

There is compelling evidence that alternatively activated macrophages are not only a vital homeostatic element in the periphery, but that microglia, and perhaps all tissue specific macrophages, also have the capacity for multiple activation states, defined by the environmental milieu in normal and disease conditions. Although considerably more work pertaining to peripheral macrophages has been accomplished, a significant effort has been made to better define microglial activation phenotypes. The notion that there are either ‘good’ or ‘bad’ activation states of microglia has lost favor. Rather, there exists a spectrum that spans several different activation types with different functions, as represented in our working model (Figure 1). By understanding the nature of microglial activation states and identifying particular induction signals for select states, we come closer to utilizing such signals as therapeutic tools in pathological conditions where detrimental polarization may contribute to disease.

## Abbreviations

AAV: adeno-associated virus; Aβ: amyloid β; APP: amyloid precursor protein; Arg1: arginase 1; CNS: central nervous system; DAMP: damage-associated molecular pattern; EAE: experimental autoimmune encephalomyelitis; ELISA: enzyme-linked immunosorbent assay; GA: glatiramer acetate; IFNγ: interferon-γ; IL-4: interleukin 4; iNOS: nitric oxide synthase; NOD: nucleotide-binding oligomerization domain; RXR: retinoid X receptor; TLR: toll-like receptor.

## Competing interests

The authors declare that they have no competing interests.

## Authors’ contribution

JDC researched the literature and drafted the manuscript. JAO and MKO critically reviewed and edited the work. All authors read and approved the final manuscript.
